# Impact of circulating miRNA-373 on breast cancer diagnosis through targeting VEGF and cyclin D1 genes

**DOI:** 10.1186/s43141-021-00174-7

**Published:** 2021-06-05

**Authors:** Noha M. Bakr, Magda Sayed Mahmoud, Reem Nabil, Hussein Boushnak, Menha Swellam

**Affiliations:** 1 Biochemistry Department, Genetic Engineering and Biotechnology Research Division, National Research Centre, Dokki, Giza, 12622 Egypt; 2 High Throughput Molecular and Genetic laboratory, Center for Excellences for Advanced Sciences, National Research Centre, Dokki, Giza, 12622 Egypt; 3 Clinical Pathology Department, National Cancer Institute, Cairo, Egypt; 4 Surgery Department, Faculty of Medicine, Ain Shams University, Cairo, Egypt

**Keywords:** Breast cancer, Clinico-pathological characteristics, VEGF, Cyclin D1, Diagnosis, MicroRNA

## Abstract

**Background:**

Breast cancer (BC) is the common primary tumor among females. Hence, there is an urgent need to improve the early prediction and diagnosis of BC. For that reason, the object of the current study is to analyze the expression levels of miRNA-373 and its target genes including vascular endothelial growth factor (VEGF) and cyclin D1 in women with BC.

**Results:**

Upregulation of miRNA-373 and its target genes was observed in BC patients followed by patients with benign breast lesions compared to downregulation in controls. There was a significant association between the expression level of miRNA-373 and all clinical features. The same associations were observed between its target genes and all clinico-pathological features except hormonal status. The correlation between miRNA-373 and both genes was significant.

**Conclusions:**

Our results prove that miRNA-373, as an oncomir, would be a vital biomarker for BC diagnosis and prognosis by targeting both VEGF and cyclin D1.

## Background

Breast cancer (BC) is the most frequently diagnosed malignant tumor in women and the principal cause of female cancer-related mortality [1]. Therefore, there is an urgent need to assess specific biomarkers of diagnostic value in order to decrease cancer-related deaths [2].

The cell cycle progression plays a crucial role in cell proliferation, and its deregulation is the most important cause of tumorigenesis [3–5]. Also, vascularization of tumor cells is pivotal in cancer initiation and progression [6]. Normal cells have a limited capability for cell division, once reaching an optimal cell density within a tissue, they will stop from proliferation, block at the gap 0 (G0) phase of the cycle, and still inactive, and this performance is owing to the response to the environmental growth inhibitory effects [7]. This cell cycle arrest mechanism, which physiologically adapted, is abnormal in tumor cells [8, 9]. In BC, anomalies of the cell cycle are frequently noted, including loss of function of retinoblastoma (Rb), decreased abundance of cyclin-dependent kinase (CDK) inhibitors like p21 and p27, and overexpression of D and E type cyclins [10, 11].

Angiogenesis is a physiological process that is the creation of novel capillaries from pre-existing blood vessels [12, 13]. The process of angiogenesis is critical not only for normal processes, but also for pathophysiological alterations like cancer development [14]. In cancer progression, the unbalance between pro- and anti-angiogenic factors causes abnormal homeostasis of the blood vessels [15]. However, some types of cancer benefit from anti-angiogenic therapies while others resist treatment, which may be due to the variation of gene expression as reported earlier [6].

miRNAs play critical roles in biological processes like cell cycle regulation, differentiation, migration, and tumor progression [16]. miRNAs, a new category of small non-coding RNA molecules, regulate the expression of their targeted genes post-transcriptionally and affect various important pathways involved in tumorigenesis [17]. A human embryonic stem cell (ESC)-specific miRNA, miRNA-373, has new tumorigenic influences through mediating the proliferation and tumorigenesis of primary human cells that harbor oncogenic mediators involving rat sarcoma (RAS) and wild-type p53 [18–20]. It could be a potential biomarker for breast cancer patients due to its differential expression and close relation with tumor invasion and metastasis in cancer cells and tissue reports [21, 22].

According to the miRTarBase (http://miRTarBase.mbc.nctu.edu.tw/), a database that covers miRNA-target interactions (MTIs), miRNA-373 targets many genes, among them are cyclin D1 and vascular endothelial growth factor (VEGF). Cyclin D1 is the protein product of the CCND1 gene, which is found on chromosome 11q13 [23]. It is the well-distinguished cell cycle regulator through binding with cdk4/6 creating a cyclin D1-cdk4/6 complex [24]. In the course of the cell cycle progression, this associate stimulates the phosphorylation and deactivation of the Rb, causing transporting cells from gap 1 (G1) phase to synthesis (S) phase [25]. When cyclin D1 deregulated (overexpressed and accumulated), it becomes an oncogene in several tumors including BC and causes several genetic alterations in regulatory proteins of the cell cycle [25]. In addition to its CDK-dependent functions, it may stimulate ER-mediated transcription, independent of estrogen, and in this way altering the estrogen response [26].

VEGF, pro-angiogenic factor and prognostic marker with numerous types of cancer including BC [27], plays a vital role in tumor progression and metastasis [28]. In BC, it plays a key role in the progression of the disease through its influences on tumor angiogenesis and through its autocrine functions in breast cancer cell migration and invasion [29].

The goal of the current study is to examine the expression levels of miRNA-373 and VEGF, and cyclin D1 in Egyptian breast cancer patients as minimal noninvasive molecular markers for diagnostic purposes, and their impact on clinical characteristics of breast cancer. To the best of our knowledge, this is the first study to investigate the effect of dysregulation of miRNA-373 on the expression levels of its target genes including VEGF as well as cyclin D1 in breast cancer patients.

## Methods

### Study design and sample processing

A total of 321 participants were enrolled in the current study; they were divided into newly diagnosed breast cancer patients (*n*=196), patients with benign breast lesions (*n*=76), and healthy volunteers as the control group (*n*=49). The inclusion criteria included those who have not received any treatment modalities or have any other malignancies. The exclusion criteria included patients with invasive lobular carcinoma (ILC) and triple-negative breast cancer (TNBC). The staging and grading classifications of BC patients [30] were obtained from their clinical sheets.

After obtaining ethical approval from the Medical Ethical Committee, and signing the informed consent, 5 ml blood was withdrawn from the enrolled individuals and divided into two tubes: one with polymer gel and clot activator that allow for further separation of the serum and stored in −80 °C for further processing to separate miRNA, and the other tube containing ethylenediamine tetraacetic acid (EDTA) for further processing of RNA separation and genes expression detection.

### miRNA and RNA isolation from human serum samples

The miRNA and mRNA isolations were performed following the instructions in the manual manufacturer’s protocols of the Qiagen miRNeasy Mini Kit (Cat number# 217004, Qiagen, Hilden, Germany) and QIAamp RNA Blood Mini kit protocol (Cat no # 52304 Qiagen, Hilden, Germany), respectively. All preparations and handling of RNA were performed in a laminar flow hood, under RNase-free conditions. The concentration and purity of the resulted miRNA and RNA were evaluated using the Nanodrop spectrophotometer (Quawell, Q-500, Quawell Technology Inc., San Jose, CA), and then stored at −80 °C until used.

### cDNA synthesis

Reverse transcription of miRNA and RNA was performed by the MiScript II reverse transcription kit (Cat number # 218160, Qiagen, Hilden, Germany) according to the manual manufacturer’s protocol in a final reaction volume of 20μl. The concentration and the purity of cDNA were measured by the Nanodrop spectrophotometer and then stored at −20 °C until used.

### Detection of miRNA and mRNA expression level by QPCR

QPCR was carried out for the detection of miRNA expression using the MiScript SYBR Green PCR primer assay (Cat number 218300, Qiagen, Hilden, Germany): miR-373 (Hs_miR-373, MS00031815), and endogenous control assay for RNU6B (Hs_RNU-2_11, MS00033740). The reaction mixture (25μl) included 12.5μl 2× QuantiTect SYBR Green PCR Master Mix, 2.5μl 10× miScript Universal Primer, 2.5μl 10× miScript Primer Assay, 4μl RNase-free water, and 2μl cDNA. For mRNA, the QuantiTect primer assays (product number 249900, Qiagen, USA) for VEGF primer assay (Hs_VEGF_1_SG, QT00013783) and cyclin D1 primer assay (Hs_CCND1_1_SG, QT00495285) and the endogenous control assay GAPDH (Hs_GAPDH_1_SG, QT00079247). The reaction mixture (25μl) included 12.5μl 2× QuantiTect SYBR Green PCR Master Mix, 2.5μl 10× QuantiTect Primer, 4μl RNase-free water, and 2μl cDNA. The PCR was performed in the QPCR system (Max3005P QPCR system; Stratagene, Agilent Technologies, CA) as follows: initial denaturation at 95°C for 15 min then 94°C for 15 s, 55°C for 30 s, and 70°C for 30 s for 40 cycles. The relative expression levels of the investigated miR-373, VEGF, and cyclin D1 were evaluated using the 2^−ΔΔCt^ method [31]. The cycle threshold (Ct) value is the number of qPCR cycles required for the fluorescent signal to cross a specified value. ΔCt was calculated by subtracting the Ct values of RNU6B from Ct miR-373 for the detection of its expression and subtracting the Ct values of GAPDH from Ct VEGF and cyclin D1 in case of gene expression analysis. ΔΔCt was calculated by subtracting the ΔCt of the control samples from the ΔCt of the cancer samples.

### Statistical analysis

Statistical analyses were carried out using the Statistical Package for Social Sciences (SPSS) software version 16.0, and *P* values were two-tailed and considered significant if *P*< 0.05. Receiver operating characteristic curve (ROC) was performed to allocate the best cutoff point that maximizes the sum of sensitivity and specificity for the tested miRNA-373, VEGF, and cyclin D1 and to detect their cutoff points and predictive values that discriminate between the cancer and non-cancer groups [32]. The Mann-Whitney *U* and Kruskal-Wallis tests were carried out for non-parametric analyses, and the one-way analysis of variance (ANOVA) and chi-square (*χ*^2^) tests were done as applicable. Correlations between investigated molecular markers were done using Spearman rank correlation.

## Results

### Demographic and clinico-pathologic data of investigated groups

As noticed in Table 1, among the entire groups, there was no significant difference reported between their ages and menopause status (*P*=0.923 and *P*= 0.059 respectively). According to the clinico-pathological criteria, patients with benign breast lesions were with follicular hyperplasia (*n*=36), fibrocystic changes (*n*=22), and intra-ductal papillomatsis (*n*=18), while those with newly diagnosed breast cancer were divided pathologically into duct carcinoma in situ (DCI) (*n*= 89, 45.4%) and invasive duct carcinoma (IDC) (107, 54.6%) and staged into stage 0–I (*n*= 57, 29.1%), stage (II) (*n*=53, 27%), stage (III) (*n*=70, 35.7%), and stage IV (*n*=16, 8.2%) and graded into grade I (*n*=64, 32.6%), grade II (*n*=58, 29.6%), and grade III (*n*=74, 37.8%). Lymph node (LN) involvements were detected in 95 of cases (48.5%), regarding hormonal status: positive estrogen receptor (ER), progesterone receptor (PgR), and human epidermal growth factor receptor (HER-2/neu) were positive in 67 (34.2%), 135 (68.9%), and 123 (62.8%), respectively.

**Table 1:** Demographic and clinico-pathologic data of the investigated groups

Parameter	Breast cancer	Benign patients	Healthy controls	***P*** value
**Number**	196	76	49	–
**Age (years, mean±SD)**	52.4 ± 9.1	52.0 ± 9.3	52.0 ± 9.3	0.923^a^
**Menopause status (no. (%))**				
Pre-menopausal	114 (58.2%)	39 (51.3%)	28 (57%)	0.059
Post-menopausal	82 (41.8%)	37 (48.7%)	21 (42.9%)	
**Tumor invasion**				
DCI	89 (45.4%)			
IDC	107 (54.6%)			
**Tumor depth (stage)**				
Stage I	57 (29.1%)			
Stage II	53 (27%)			
Stage III	70 (35.7)			
Stage IV	16 (8.2%)			
**Tumor histological grade**				
Grade I	64 (32.6%)			
Grade II	58 (29.6%)			
Grade III	74 (37.8%)			
**Lymph node invasion**				
Negative	101 (51.5%)			
Positive	95 (48.5%)			
**ER**				
Negative	67 (34.2%)			
Positive	129 (65.8%)			
**PgR**				
Negative	61 (31.1%)			
Positive	135 (68.9%)			
**HER-2/neu status**				
Negative	73 (37.2%)			
Positive	123 (62.8%)			

*P* > 0.05 is considered non-significant; *P* < 0.05 is considered significant

*DCI* duct carcinoma in situ, *IDC* invasive duct carcinoma, *ER* estrogen receptor, *PgR* progesterone receptor, *Her2/neu* human epidermal growth factor receptor-2

^a^Data were represented as mean ±SD

### Expression of miRNA-373, VEGF, and cyclin D1 among the studied groups

**Fig. 1:**
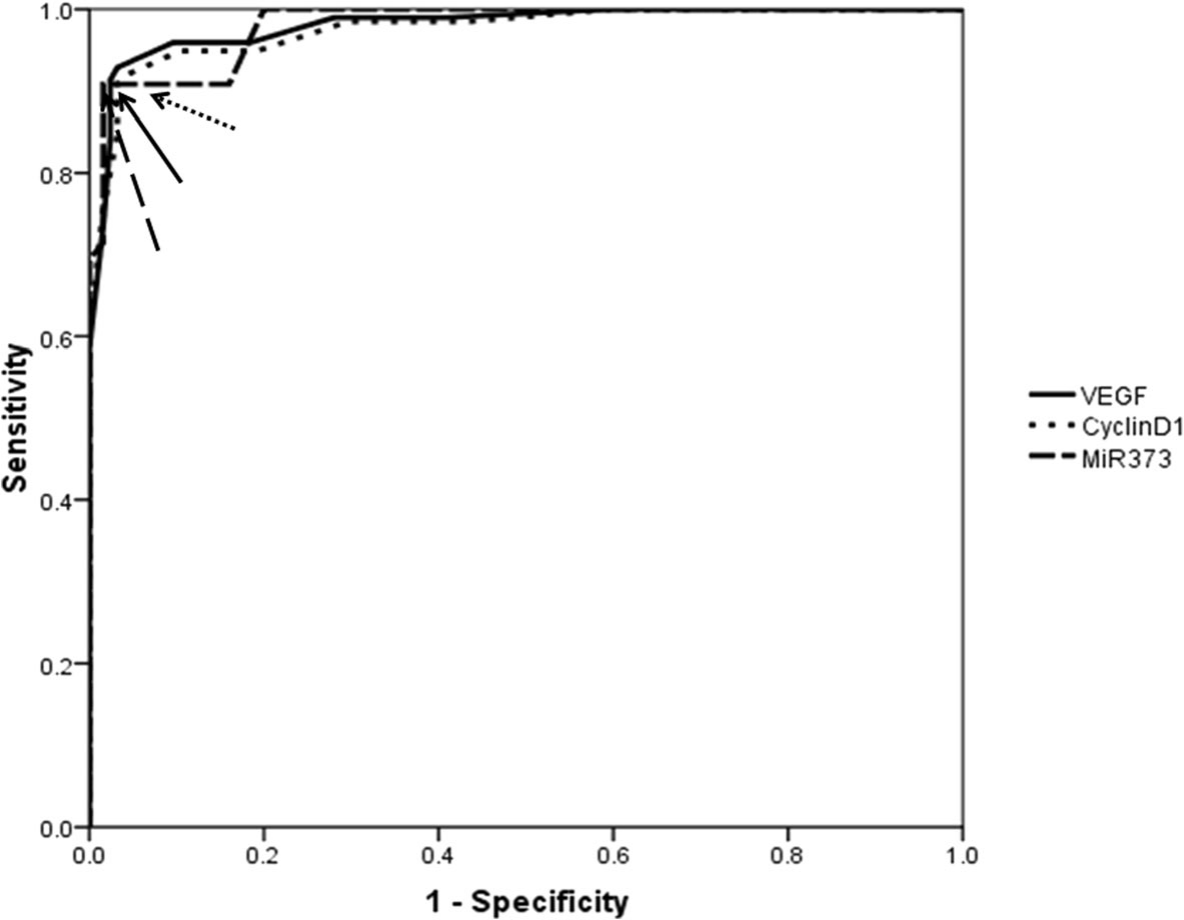
Receiver operating characteristic curve for investigated markers. Arrows donate to the cut value for investigated markers: for VEGF, the cutoff was 38 ng/ml with sensitivity at 92.35% and specificity at 96.8% at standard error 0.0077 and CI% 0.955–0.991, area under the curve 0.978; for cyclin D1, the cutoff was 38 ng/ml with sensitivity at 91.8% and specificity at 96% at standard error 0.008 and CI% 0.952–0.989, area under the curve 0.975; and for miR-373, the cutoff was 360 ng/ml with sensitivity at 90.8% and specificity at 98.4% at standard error 0.007 and CI% 0.958–0.992, area under the curve 0.98

As reported in Table 2, the mean rank level for miRNA-373 and investigated genes, VEGF and cyclin D1, reported a significant increase among primary breast cancer patients followed by benign cases while their expression were the lowest in healthy individual cases (*P*< 0.0001). Similarly, by plotting the ROC curve for discrimination between cancer and non-cancer groups (benign and healthy) (Fig. 1), a significant difference was reported (*P*< 0.0001) between the three groups as the frequency of the positivity rate (> cutoff point) was superior for breast cancer cases at 91.3%, 92.9%, and 91.8% for miRNA-373, VEGF, and cyclin D1, respectively.

**Table 2:** Frequency distributions of investigated markers among different studied groups

Groups	Markers								
	VEGF (fold change)			Cyclin D1 (fold change)^a^			miR-373 (fold change)^a^		
	Mean rank^a^	≤ 38^b^	> 38	Mean rank^a^	≤ 38	> 38	Mean rank^a^	≤ 360	> 360
Healthy individuals	32.86	49 (100%)	0 (0%)	30.92	49 (100%)	0 (0%)	27.04	49 (100%)	0 (0%)
Benign lesions	88.78	73 (96.1%)	3 (3.9%)	91.59	72 (94.7%)	4 (5.3%)	92.57	75 (98.7%)	1 (1.3%)
Primary breast cancer	221.05	14 (7.1%)	182 (92.9%)	220.43	16 (8.2%)	180 (91.8%)	221.03	17 (8.7%)	179 (91.3%)

^a^Analysis using non-parametric mean rank test by Kruskal-Wallis test (as to calculate the average of the ranks for the marker among the enrolled groups)

^b^Chi-square test with statistical significance at *P*< 0.0001

### Prognostic presentation of investigated markers among the breast cancer group

The mean rank levels for miRNA-373, VEGF, and cyclin D1 were investigated regarding clinico-pathological factors (age, menopausal status, pathological types, clinical stage, histological grading, and hormonal receptor status) among the primary breast cancer group. No significant difference (*P*>0.05) was reported between investigated markers and age or menopausal status.

**Fig. 2:**
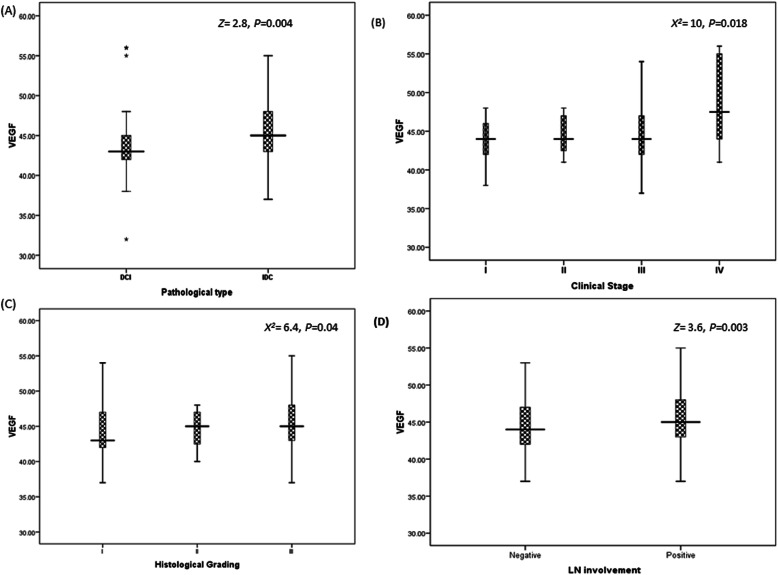
Relation between miR-373 and clinico-pathological features. **a** Pathological types (DCI vs IDC) at *X*^*2*^=3.5 (*P*<0.001). **b** Clinical stages (stage I to IV) at *X*^*2*^=10 (*P*=0.012). **c** Histological grading (grade I to III) at *X*^*2*^=10 (*P*=0.006). **d** Lymph node involvement (negative involvement vs positive involvement) (*X*^*2*^=4.43, *P*<0.001). *X*^2^ resembles the chi-square test between investigated variables

miRNA-373 reported significant difference as shown in Fig. 2a with pathological types as their the mean rank levels were 111.48 in IDC as compared to 82.89 for DCI (*P*<0.001), also with clinical stages (Fig. 2b) 83.02, 107, 68, 96.42, and 128.97 with stages I, II, III, and IV, respectively (*P*=0.012). Regarding histological grading, a significant level (*P*=0.006) is reported at Fig. 2c as miRNA-373 expression level was elevated in advanced grade as compared to low ones. A significant difference was reported with lymph node status as lymph node involvement reported high mean rank miRNA-373 (117.7) as compared to negative LN involvement (80.4) (*P*<0.0010) as plotted in Fig. 2d. The relation between miRNA-373 and hormonal status is shown in Table 3.

**Table 3:** Mean rank level of VEGF, cyclin D1, and miR-373 among the primary breast cancer group (*n*=196)

Characteristics	VEGF	Cyclin D1	miR-373
**ER status**			
Negative (*n*=20)	84.8	90	82
Positive (*n*=76)	113	107	115
	*Z*= 3.5, *P*<0.001	*Z*= 2.16, *P*= 0.031	*Z*= 4, *P*<0.001
**PgR status**			
Negative (*n*=35)	86	97	91
Positive (*n*=61)	104	101	114
	*Z*= 3.4, *P*= 0.037	NS	*Z*=2.6, *P*=0.008
**HER-2/neu status**			
Negative (*n*=37)	95.8	99.4	91
Positive (*n*=51)	100.08	98	110
	NS	NS	*Z*=2.28, *P*=0.022

**Fig. 3:**
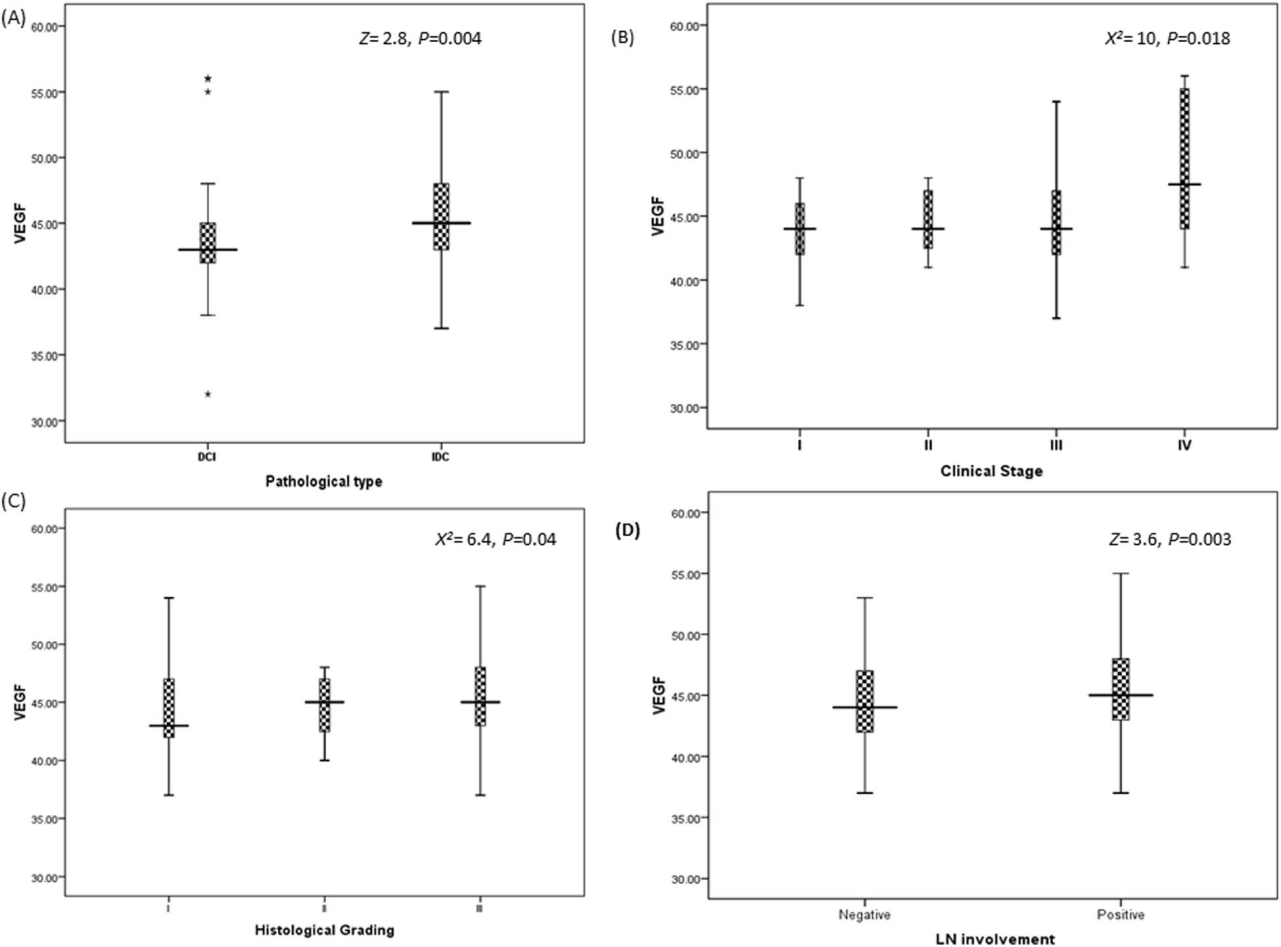
Relation between VEGF and clinico-pathological features. **a** Pathological types (DCI vs IDC) at *Z*=2.8 (*P*=0.004). **b** Clinical stages (stage I to IV) at *X*^*2*^=10 (*P*=0.018). **c** Histological grading (grade I to III) at *X*^*2*^=6.4 (*P*=0.04). **d** Lymph node involvement (negative involvement vs positive involvement) (*Z*=3.6, *P*=0.003). Statistical analysis was done using the non-parametric test; as for two variables, the Mann-Whitney *U* test (*Z*-value) was used; and for more than two variables, the Kruskal-Wallis *H* test (*X*^*2*^ test) was used

For VEGF, a significant level was reported between VEGF gene expression and pathological type as its level was significantly increased in IDC as 111.12 as compared to DCI which equals 86 (*P*=0.004) (Fig. 3a); also, a significant difference was reported with clinical stages (Fig. 3b); the mean rank levels reported 103.8, 101.37, 86.6, and 134.3 with stages I, II, III, and IV, respectively (*P*=0.018), similarly with grading as presented in Fig. 3c with mean ranks as 85.5, 97.9, and 110 for grades I, II, and III, respectively (*P*=0.04). LN status reported a significant difference with VEGF expression level as the mean rank for negative LN was 86.9 vs positive LN was 110.8 (*P*=0.003) (Fig. 3d). The impact of VEGF expression with hormonal receptor status is represented in Table 3.

**Fig. 4:**
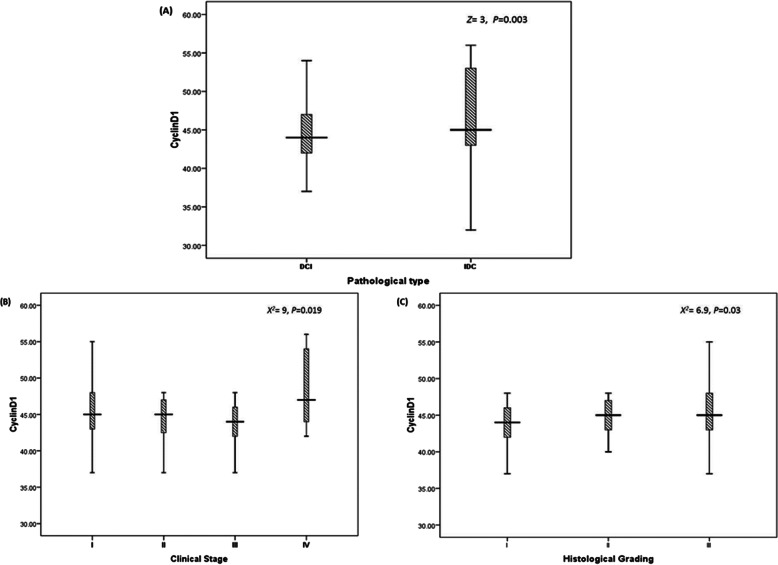
Relation between cyclin D1 and clinico-pathological features. **a** Pathological types (DCI vs IDC) at *Z*=3 (*P*=0.003). **b** Clinical stages (stage I to IV) at *X*^*2*^=9 (*P*=0.019). **c** Histological grading (grade I to III) at *X*^*2*^=6.9 (*P*=0.03). Statistical analysis was done using the non-parametric test; as for two variables, the Mann-Whitney *U* test (*Z*-value) was used; and for more than two variables, the Kruskal-Wallis *H* test (*X*^*2*^ test) was used

Regarding cyclin D1, a significant difference was reported with pathological classification as its expression elevated in IDC to be 111.67 as compared to 87.47 for DCI (*P*=0.003) (Fig. 4a). Also, the mean rank level reported significant difference with clinical stages as well; the mean rank levels reported in Fig. 4b 103.8, 96.79, 86.6, and 132 with stages I, II, III, and IV, respectively (*P*=0.019), also significant level with histological grading as 85.6, 97, and 110,8 for grades I, II, and III (*P*=0.03) as shown in Fig. 4c. The expression of cyclin D1 with hormonal status is represented in Table 3.

### Correlation between miRNA and investigated genes

The correlation between miRNA-373 and both genes were significant (*R* = 0.352, *P*< 0.001) with VEGF and (*R* =0.365, *P*< 0.001) with cyclin D1. Also, the two genes were significantly correlated with each other (*R* =0.344, *P*< 0.001).

## Discussion

Recently, evidence proposes that miRNAs have significant roles in various cellular events, and their expression patterns might be precious markers in the diagnosis of several cancer types and patient outcomes [33, 34]. To the best of our knowledge, the present study that was performed on the Egyptian cohort is the first which assesses the expression level of circulating miRNA-373 and its target genes including VEGF and cyclin D1 in blood specimens of BC patients.

There are several literatures demonstrating the oncogenic role of miRNA-373 through targeting various genes in several types of human cancer [35–40]. Nevertheless, to date, functions of miRNA-373 in BC remain debated [20], which motivated us to examine the role of miRNA-373 in BC; according to our results, high expression levels of circulating miRNA-373 were reported in primary breast cancer patients followed by benign cases compared to the lower expression levels in healthy volunteers. These results were consistent with Eichelser et al. [41]. Based on these findings, it seems that miRNA-373 and its target genes were valuable molecular biomarkers in the early prediction and diagnosis of BC, and in the discrimination between cancer and non-cancer cases.

A significant association was detected between miRNA-373 expression level and unfavorable prognostic factors for BC which agree with the previous report of Chen et al. [42], and this may be due to its role to promote BC invasion and metastasis by inhibiting the protein expression of the cell surface marker cluster of differentiation 44 (CD44) [21]. Moreover, it was able to trigger the Wnt/β-catenin-signaling pathway, which is implicated in both stem cell maintenance and tumorigenesis [43].

Angiogenesis plays a key role in the development of tumor growth and metastasis, VEGF is one of these angiogenic markers, and it has been overexpressed in breast cancer as compared to the other investigated groups which agreed with previous reports [44–46]. The association of the expression level of the VEGF gene with the clinico-pathological characteristics of BC was examined, and VEGF expression level was associated with IDC, high staging, high grading, lymph node metastasis, and finally hormonal status except HER2-neu status, which directs the importance of VEGF as both diagnostic and prognostic marker. Using of anti-angiogenic drugs as anti-VEGF drugs (Avastin (bevacizumab) or ranibizumab (Lucentis)) has effectually proved to prevent tumor growth specifically in combination with chemotherapy or immunotherapy [47]. As previously reported, the combination of bevacizumab with conventional chemotherapy can increase both survival and response to treatment in patients with different types of cancer [48–50].

Cyclin D1 expression was significantly increased in breast cancer patients as compared to both benign and control individuals; these findings were in line with Elsheikh and his colleagues [44] as they noted that a strong association between CCND1 amplification and its protein expression (cyclin D1) in breast cancer. Also, Ravikumar and Ananthamurthy [51] showed that high cyclin D1 expression was identified 67.5% in ductal carcinoma. Moreover, Hartel et al. [52] reported that cyclin D1 expression may serve as a marker for more biologically aggressive triple-negative breast cancer. Additionally, Ortiz et al. [53] showed that high cyclin D1 expression was identified in 67.5% in ductal carcinoma and 52% of invasive breast cancers. In respect to the association of cyclin D1 expression level with the clinico-pathological features of BC, our findings showed that the overexpression of cyclin D1 was associated with IDC, high staging, high grading, lymph node metastasis, and finally hormonal status except HER2-neu status. Cyclin D1 expression was correlated with ER expression [54] in agreement with our findings. These results indicate the vital role of cyclin D1 in estrogen-induced breast cancer, as estrogen action is induced through transcriptional activation of cyclin D1 and cellular-Myc (c-Myc) [55–57].

The relation between miRNA-373 and the studied genes was investigated and reported significant correlation which agreed with the previous study reported a relation between the overexpression of miRNA-373 and VEGF gene in cancerous tissues that emphasized the fact that miRNA-373 acts as an essential factor in tumorigenesis and metastasis [36]. In contrast to our results, Tavazoie et al. [22] reported that miRNA-373 is a metastasis-promoting factor through inhibiting the expression of cyclin D1.

## Conclusion

miRNA-373 expression is considered an oncomir that is related to VEGF and cyclin D1 expression, and all were related to tumorigenesis. Hence, their detection in circulating blood may aid in better early diagnosis of breast cancer, and prognosis of such disease. Thus, it is recommended to investigate these findings on a large number of cases as their uses in clinical practice may aid in the early detection of breast cancer and help in targeted therapy.

## Data Availability

Not applicable.

## Abbreviations

BC: Breast cancer
VEGF: Vascular endothelial growth factor
QPCR: Quantitative polymerase chain reaction
G0: Gap 0
CDK: Cyclin-dependent kinase
pRb: Retinoblastoma protein
ESC: Human embryonic stem cell
RAS: Rat sarcoma
MTIs: miRNA-target interactions
ILC: Invasive lobular carcinoma
TNBC: Tiple-negative breast cancer
EDTA: Ethylenediamine tetraacetic acid
Ct: Cycle threshold
SPSS: Statistical Package for Social Sciences
ROC: Receiver operating characteristic curve
ANOVA: One-way analysis of variance
SE: Standard error
DCI: Duct carcinoma in situ
IDC: Invasive duct carcinoma
LN: Lymph node
ER: Estrogen receptor
PgR: Progesterone receptor
HER-2/neu: Human epidermal growth factor receptor
CD44: Cluster of differentiation 44
c-Myc: Cellular-Myc
G1: Gap 1
S: Synthesis phase
